# MicroRNA‐661 modulates redox and metabolic homeostasis in colon cancer

**DOI:** 10.1002/1878-0261.12142

**Published:** 2017-11-06

**Authors:** Marta Gómez de Cedrón, Rebeca Acín Pérez, Ruth Sánchez‐Martínez, Susana Molina, Jesús Herranz, Jaime Feliu, Guillermo Reglero, Jose Antonio Enríquez, Ana Ramírez de Molina

**Affiliations:** ^1^ Precision Nutrition and Cancer Program Molecular Oncology and Nutritional Genomics of Cancer Group IMDEA Food Institute, CEI UAM + CSIC Madrid Spain; ^2^ Functional Genetics of the Oxidative Phosphorylation System Spanish National Cardiovascular Research Centre (CNIC) Madrid Spain; ^3^ Medical Oncology La Paz University Hospital (IdiPAZ‐UAM) Madrid Spain

**Keywords:** bioenergetics, colon cancer, metabolomics, miR, oxidative stress

## Abstract

Cancer cell survival and metastasis are dependent on metabolic reprogramming that is capable of increasing resistance to oxidative and energetic stress. Targeting these two processes can be crucial for cancer progression. Herein, we describe the role of microRNA‐661 (miR661) as epigenetic regulator of colon cancer (CC) cell metabolism. MicroR661 induces a global increase in reactive oxygen species, specifically in mitochondrial superoxide anions, which appears to be mediated by decreased carbohydrate metabolism and pentose phosphate pathway, and by a higher dependency on mitochondrial respiration. MicroR661 overexpression in non‐metastatic human CC cells induces an epithelial‐to‐mesenchymal transition phenotype, and a reduced tolerance to metabolic stress. This seems to be a general effect of miR661 in CC, since metastatic CC cell metabolism is also compromised upon miR661 overexpression. We propose hexose‐6‐phosphate dehydrogenase and pyruvate kinase M2 as two key players related to the observed metabolic reprogramming. Finally, the clinical relevance of miR661 expression levels in stage‐II and III CC patients is discussed. In conclusion, we propose miR661 as a potential modulator of redox and metabolic homeostasis in CC.

**Abbreviations**
AcCoA: Aacetyl‐CoA
BNGE: blue native gel electrophoresis
BRR: basal respiration rate
CC: colon cancer
DFS: disease‐free survival
DHAP: dihydroxyacetone phosphate
ECAR: extracellular acidification rate
EMT: epithelial to mesenchymal transition
ETC: electron transport chain
FAO: fatty acid oxidation
FFPE: formalin‐fixedparaffin‐embedded
H6PD: hexose‐6‐phosphate dehydrogenase
HR: hazard ratio
IDH1: isocitrate dehydrogenase
ME: malic enzyme
miR661: microRNA‐661
MRR: maximal respiration rate
OCR: oxygen consumption rate
OS: overall survival
PEP: phosphoenolpyruvate
PKM2: pyruvate kinase M2
PPP: pentose phosphate pathway
Pyr: pyruvate
ROS: reactive oxygen species
SC: supercomplexes
SO^‐^: superoxide anions
TCA: tricarboxylic acid cycle
Ψm: mitochondrial membrane potential

## 1 Introduction

Tumor cells reprogram their energetic metabolism to support rapid and uncontrolled growth. The capacity to manage and overcome associated metabolic stress is crucial for cancer cell survival and metastasis (Jeon *et al*., 2012).

It is well known that oncogenic transformation alters cellular metabolism to facilitate cell proliferation, but less is known about the metabolic changes that promote epithelial to mesenchymal transition (EMT) and the EMT‐associated metabolic reprogramming.

Epithelial‐to‐mesenchymal transition is a conserved morphogenetic program characterized by the loss of the epithelial phenotype and the gain of mesenchymal features (Nieto and Cano, 2012). Epithelial cells lose cell polarity and cell‐to‐cell adhesions, gaining a mesenchymal morphology with migration and invasion capabilities. These properties promote metastasis and the development of several neoplasias (Gupta and Massague, 2006), including colorectal cancer, one of the most common cancers worldwide and the second most common malignant tumor in Europe, killing 230 000 people each year (http://www.europacolon.com).

MicroRNAs (miRs) are small endogenous noncoding RNAs (∼22 nucleotides) involved in the posttranscriptional regulation of gene expression. miRs bind to complementary regions within the 3′UTR of messenger RNAs (mRNA) promoting their degradation and/or inhibiting translation (Bartel, 2004). miRs have an important role in nearly all biological processes and aberrant miRNA expression is associated with many diseases, including cancer (Gomez de Cedron and Ramirez de Molina, 2016). Importantly, the expression levels of specific miRs have been correlated with clinicopathological variables acquiring a diagnostic and prognostic value (Chen *et al*., 2016; Kingham *et al*., 2017). For these reasons, interest in the role of miRs in cancer has increased significantly in the past few years (Kosaka *et al*., 2010).

Due to the promiscuous activity of miRs towards different targets, the very same miR may promote or inhibit tumorigenesis in different cell specific scenarios. A clear example of this situation is miR661. The final output of the effect of this miR relies not only on the specific cancer cell type, but also on multiple players associated to the cell biology: oncogenes, tumor suppressors, metabolites and/or oxidative stress associated molecules. In this sense, depending on p53 status, miR661 may suppress (p53‐wild‐type) or promote (p53‐mutated) cancer aggressiveness by a direct effect on mdm2 and mdm4 (Hoffman *et al*., 2014). In a model of Snail1‐induced EMT in breast cancer cells, Vetter and collaborators identified miR661 as a key Snail1‐induced miR required for efficient invasion (Vetter *et al*., 2010). In contrast, Reddy *et al*. (2009) reported that miR661 inhibited the expression of metastatic tumor antigen 1 (MTA1) in invasive breast cancer cells and reintroduction of this miR was able to inhibit motility, invasiveness, and tumorigenesis. However, this controversy disappears when one takes into account that, in the first work, Snail drives the EMT phenotype and miR661 is an associated player, whereas in the second work, miR661, by targeting MTA1, induces the opposite effect. In ovarian cancer, miR661 promotes cell proliferation by targeting INPP5J (Zhu *et al*., 2015), but in gliomas it inhibits cell proliferation, migration and invasion by targeting hTER (Li *et al*., 2015).

To elucidate the role of miR661 in colon cancer (CC) we present an integrative approach, including metabolomics, analysis of cell bioenergetics, and molecular targets identification. MicroR661 is sufficient to induce EMT in non‐metastatic CC cells and an increase in reactive oxygen species (ROS), partially mediated by decreased carbohydrate metabolism and pentose phosphate pathway, as well as a higher dependency on mitochondrial respiration. miR661 diminishes the tolerance to metabolic stress in both non‐metastatic and metastatic CC cells and we propose hexose‐6‐phosphate dehydrogenase (H6PD), and pyruvate kinase M2 (PKM2) as two major players on miR661‐induced metabolic reprogramming. Finally, high levels of miR661 were correlated with poorer prognosis in stage‐II CC, consistent with EMT and invasiveness promotion upon miR661 overexpression in non‐metastatic CC cells. In contrast, high expression levels of miR661 in stage‐III CC patients were correlated with a better clinical outcome, in accordance with miR661 overexpression in metastatic CC cell results. We conclude that miR661 may play a dual role in CC, being involved in the aggressiveness of the tumor at the onset of the metastasis but offering a potential therapeutic window against invasive tumors. Based on these findings, we propose miR661 as a potential modulator of redox and metabolic homeostasis in CC.

## 2 Materials and methods

### 2.1 Cell culture

The colon cancer cell lines DLD1 and SW620, and HEK‐293T cells were obtained from the American Type Culture Collection (ATCC, Manassas, VA, USA), and maintained under standard conditions. All cell lines were authenticated by microsatellite genotyping. As described by Lothe and colleagues, both colon cancer cell lines present mutated TP53 (DLD1: S241F and SW620: R273H;P309S) (Ahmed *et al*., 2013).

### 2.2 Reagents

N‐acetylcysteine was from Sigma‐Aldrich (Sigma‐Aldrich, St. Louis, MO, USA).

### 2.3 Lentivirus‐mediated stable overexpression of miR661

HEK 293T cells were transfected using Lipofectamine 2000 (Life Technologies, Carlsbad, CA, USA) with a lentiviral vector expressing miR661 (DNA 2.0, Menlo Park, CA, USA) and packaging plasmids (Addgene, Cambridge, MA, USA). DLD1 and SW620 cells were infected with the collected supernatants in the presence of polybrene (4 μg·mL^−1^) as coadjutant. Finally, stable cells were selected with puromycin for 2 weeks.

### 2.4 Quantitative real‐time PCR

Total RNA was extracted using Tri Reagent (Sigma). Briefly, 1 μg of total RNA was reverse‐transcribed using the High Capacity RNA‐to‐cDNA Master Mix system (Life Technologies). Table S2 displays the list of the primers used for qRTPCR.

miR661 and gene‐expression assays were performed in a HT‐7900 Fast Real time PCR. Values were normalized using *U6snRNA* (for miR661) and *GAPDH* expression. Relative expression was calculated by the 2^−∆∆Ct^ method.

### 2.5 Invasion assays

Invasion assays were performed with BD Biosciences Matrigel™ (Madrid, Spain) invasion chambers following manufacture indications. Images were captured using an Olympus CKX41 microscope (Olympus, Tokyo, Japan), with a 20× objective and analysis getit software (Olympus).

### 2.6 Dual‐luciferase assays (cloning and co‐transfection)

Short sequences from the 3′UTR of *PKM (ENST00000335181)*: wt‐short 3′ UTR *PKM* and mutated (mut)‐short 3′ UTR *PKM,* and *H6PDH (ENSG00000049239)*: wt‐short 3′UTR *H6PDH* and mut‐short 3′ UTR *H6PDH*, were cloned into psiCheck2 (DNA 2.0, Menlo Park, CA, USA). HEK‐293T cells (*n =* 50 000) were transfected using Lipofectamine^®^ 2000 (Invitrogen, Madrid, Spain) accordingly to the manufacturer's recommendations. For a directional cloning, *Xho*I and *Not*I restriction sites were included in all constructs. (Fig. S4; seed sequence binding sites are underlined and point mutations in the seed sequence are shown in bold).

To assess the direct effect of miR661, 100 ng of psiCheck2‐3′‐UTR‐short‐wt‐*PKM*, 3′‐UTR‐short‐mut‐*PKM*, 3′‐UTR‐short‐wt‐*H6PDH* or 3′‐UTR‐short‐mut‐*H6PDH* were co‐transfected with 30 nM of LNA‐miR661 in HEK293T cells. The translational repression of *PKM or H6PDH* upon miR661 binding was determined after transfection using the Dual‐Luciferase Reporter Assay System (Promega Biotech Ibérica S.L., Madrid, Spain). Relative luciferase activity (Renilla luminescence/Firefly luminescence) was represented.

### 2.7 Lentivirus‐mediated transient overexpression of PKM2 and H6PD

HEK293T cells were transfected using Lipofectamine^®^ 2000 (Life Technologies) with lentiviral vectors expressing *PKM2* and/or *H6PDH* (DNA 2.0) along with a set of packaging plasmids (Addgene). DLD1 cells were infected with supernatants produced upon 48‐h post‐transfection in HEK293T cells and 4 μg·μL^−1^ polybrene (Merck Millipore, Madrid, Spain) as coadjutant. Cells were collected 48 h post‐infection for RNA and cell bioenergetics assays.

### 2.8 List of antibodies for western blot

Primary antibodies were N‐cadherin (333900, Cell Signaling Technology Europe Invitrogen, Leiden, the Netherlands); AMPK‐α (Cell Signaling #2532); P‐AMPK‐α (Thr172) (40H9, Cell Signaling #2535); GSK‐3β (27C19, Cell Signaling #9315); P‐GSK‐3 α/β(Ser21/9) (Cell Signaling #9331); PKM2 (Cell Signaling #3198); H6PD (C‐10: sc‐377180). β‐actin or vinculin were used as a loading controls as indicated.

### 2.9 l‐lactate quantification

l‐lactate quantification was done using Cayman′s Glycolysis cell‐based assay (Cayman, Ann Arbor, MI, USA, 600450) (*n* = 3).

### 2.10 Assays of reactive oxygen species

The intracellular levels of H_2_O_2_ and $O_{2}^{-}$ were measured with H2DCFDA (2,7′‐dichlorodihydrofluorescein diacetate) and MitoSOx Red (Invitrogen Molecular Probes, Madrid, Spain; M36008), respectively. The membrane potential was assayed by TMRN staining. Briefly, 10^5^ cells were seeded in a 12‐well plate and treated with the probes for 30 min. The cells were then washed with PBS and collected as single cell suspensions. PI staining was done to discriminate dead cells. Fluorescence was detected by flow cytometry.

### 2.11 Global metabolomic profile

DLD1‐miR661 and DLD1‐Control cells were prepared as indicated by Metabolon Inc. for global metabolomic analysis (Reitman *et al*., 2011). A combination of GC‐MS and LC‐MS methods was used, and each metabolite amount was normalized to total protein amount of the individual cell pellets (*n* = 4). Raw data were extracted, peak‐identified and QC‐processed using Metabolon's hardware and software. Compounds were identified by comparison with library entries of purified standards or recurrent unknown entities.

### 2.12 Extracellular flux analysis of oxygen consumption rate and extracellular acidification rate

Mitochondrial respiration and glycolytic function were analyzed using XF Cell Mito Stress Test kit and XF Glycolysis Stress kit standard assays (Seahorse Biosciences Agilent Technologies, Madrid, Spain, XF^e^96). Cell density and oligomycin or FCCP concentration were first optimized for XF Glycolysis and XF Mito Stress, respectively. For the analysis of the glycolytic function cells were maintained overnight in low glucose (5 mm) before running XF Glycolysis Stress. The following day, the medium was changed to a medium without glucose to starve the cells for 1 h (six replicates per condition, three independent experiments). Representative experiments are shown.

### 2.13 BNGE characterization of supercomplexes

Mitochondrial membranes were isolated as described by Nijtmans *et al*. (2002) and supercomplexes were solubilized with digitonin (1% final concentration). Respiratory complexes were resolved in concentrations of 3–13% by Blue Native Gel Electrophoresis (Wittig *et al*., 2006). After electrophoresis, proteins were electroblotted onto PVDF filters and sequentially probed with specific antibodies.

### 2.14 Statistical analysis

Differences between mean values were analyzed with two‐tailed Student's t‐tests. Data with *P* < 0.05 were considered significant and statistically significant differences are shown with asterisks (*P < 0.05; **P < 0.01; ***P < 0.001).

A total of 136 CRC stage II and 81 CRC stage III samples of patients from La Paz University Hospital were enrolled in the study. Formalin‐fixed, paraffin‐embedded (FFPE) samples were obtained with the patient's consent and with the approval of the human research Ethics Review Committee of La Paz University Hospital (HULP‐PI‐1452). Clinical‐histopathological data of patients were prospectively collected by clinical history and were confirmed by oncologists involved in this study. To estimate the overall survival (OS) and disease‐free survival (DFS), the Kaplan–Meier method was used. The log‐rank test for univariate Cox regression analysis was performed to test the association between DFS and gene expression. Hazard ratios (HR) and 95% CI were calculated from the Cox regression model. The expression data of the miR661 was categorized in two categories, high and low, selecting one cut‐point based in the minimum *P*‐value of the log‐rank test. The statistical analyses were performed using r statistical software version 2.15 (www.r-project.org).

## 3 Results

### 3.1 miR661 overexpression confers EMT and invasive properties to non‐metastatic CC cells

We first investigated the role of miR661 in the EMT properties of non‐invasive CC cells. For this purpose, we analyzed miR661 expression levels in a panel of CC cell lines. Among them, we selected the DLD1 non‐metastatic cell line, whose miR661 expression levels were lower than that of primary colon cell lines (Ccd18‐Co, CCD841) (Fig. S1). We generated a stable DLD1 cell line overexpressing miR661 by transduction with a lentiviral vector (DLD1‐miR661). An empty lentiviral vector was used to generate Control DLD1 cell line (DLD1‐Control).

**Figure 1:**
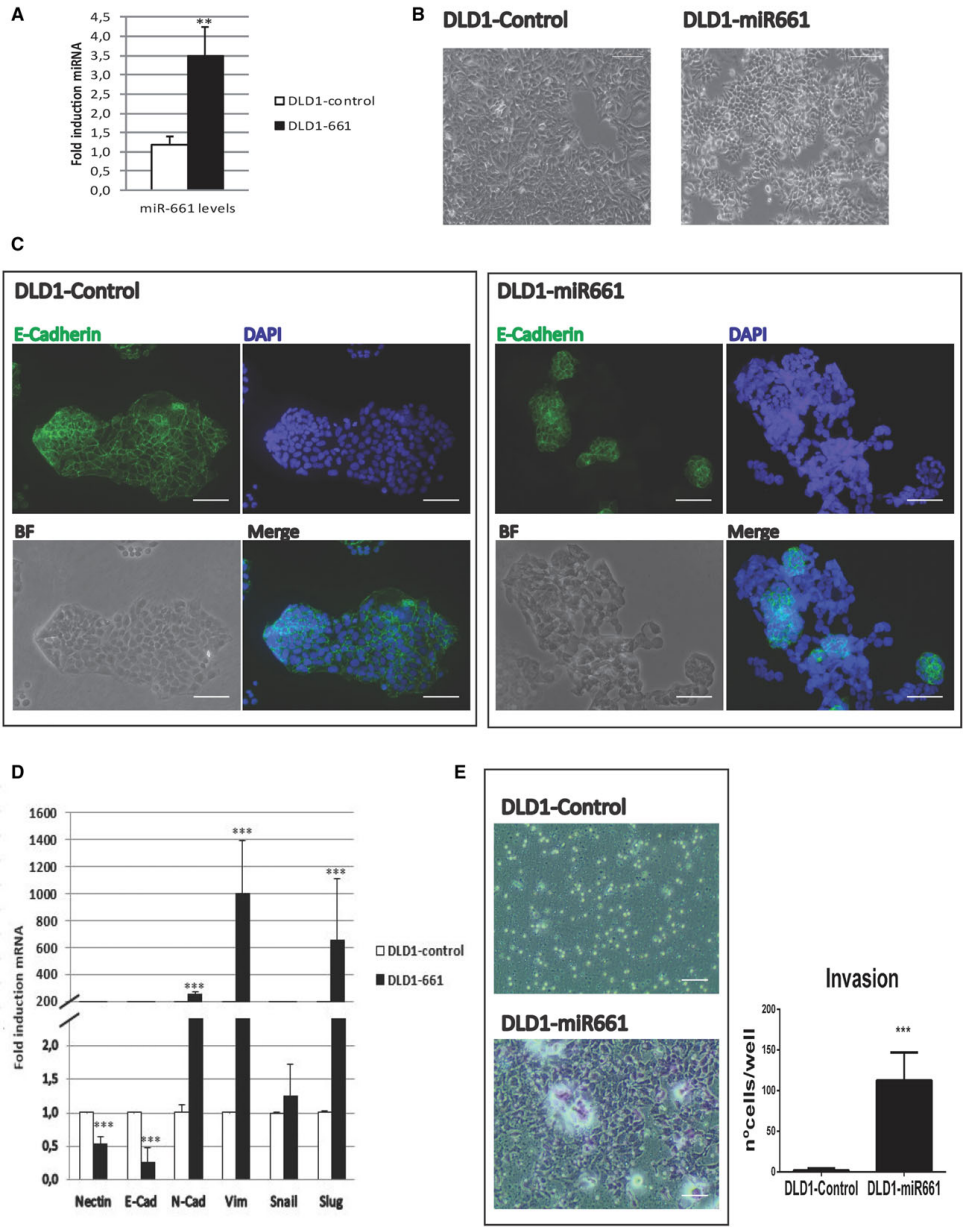
MicroR661 overexpression induces EMT in DLD1 colon cancer cell line. (A) Stable cell line overexpressing miR661 was generated using lentiviral transduction and expression levels were measured by RT‐QPCR. (B) Representative phase contrast images showing atypical morphology of DLD1‐miR661 cells compared with DLD1‐Control cells. Scale bars: 100 μm. (C) Representative immunofluorescence images of E‐cadherin (green) cellular distribution of DLD1‐miR661 and DLD1‐Control cells. Nuclei were stained with DAPI (blue) and equivalent phase contrast images were taken. Scale bars: 50 μm. (D) RT‐QPCR analysis of epithelial (*E‐Cadherin*) and mesenchymal genes (*N‐Cadherin*,* Vimentin, Snail* and *Slug*) for DLD1‐miR661 compared with levels in DLD1‐Control cells. (E) Representative images of Boyden chamber transwell assay of invasion through Matrigel. After 48 h, cells were fixed and stained with crystal violet. Scale bars: 100 μm. MicroR661 did not induce EMT or invasion properties in any metastatic DLD1 colon cancer cell line. Experiments in (A) (C), (D) and (E) were performed in triplicate (*n* = 3). Results represent the mean ± SD. **P < 0.01, ***P < 0.001.

miR661 levels were measured to ensure that stable expression of the construct was achieved (Fig. 1A shows significant increased levels of miR661 in DLD1‐miR661 compared to DLD1‐Control cells). Unlike DLD1‐Control cells, DLD1‐miR661 cells displayed morphological changes resembling an EMT phenotype (Fig. 1B). We next analyzed the expression of the epithelial marker E‐cadherin by immunofluorescence. As shown in Fig. 1C, membrane‐associated localization of E‐cadherin was disrupted in most of the cells.

We analyzed by qRTPCR the expression of epithelial and mesenchymal markers related to EMT. First, we confirmed the effect of miR661 on the downregulation of *Nectin1*, a described miR661 target (Vetter *et al*., 2010). As shown in Fig. 1D, overexpression of miR661 diminished the expression of the epithelial marker *E‐cadherin*. In contrast, miR661 increased the expression levels of the EMT‐associated genes *N‐cadherin*,* Slug* and *Vimentin*, which are normally not expressed in the markedly epithelial DLD‐1 cells.

The acquisition of EMT and invasive properties is crucial for metastasis formation and cancer progression. As illustrated in Fig. 1E, when miR661 was overexpressed, poorly invasive DLD1 cells gained the ability to invade through Matrigel. This indicates that miR661 is sufficient to induce EMT properties in DLD1 non‐metastatic colon cancer cells.

### 3.2 miR661 induces an increase of SO^·^ anions and high Ψm at mitochondria

We hypothesized that miR661 may target, in addition to EMT‐related targets, some other metabolic targets that will affect cell homeostasis. Production of reactive oxygen species (ROS) has been extensively described to drive EMT‐associated pathways (Gupta *et al*., 2012). Reactive oxygen species (ROS) produced as byproducts of metabolism or as a component of ROS‐dependent signaling processes, have emerged as key mediators of cancer initiation and development (Costa *et al*., 2014).

**Figure 2:**
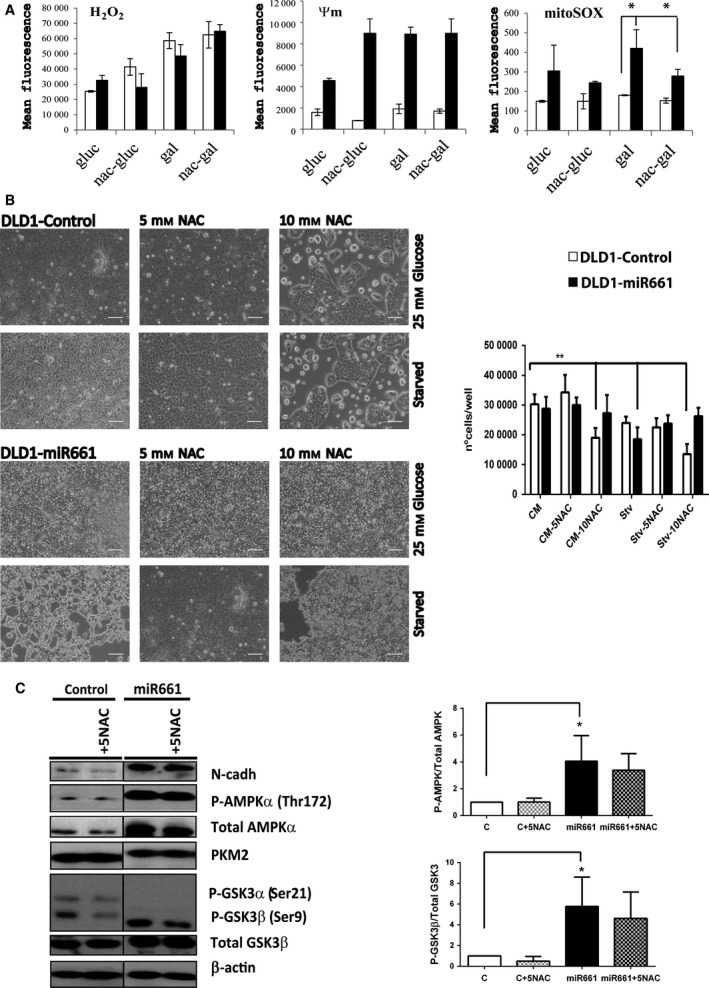
MicroR661 leads to an increase of SO ^·^ and mitochondrial membrane potential (Ψm) in DLD1 colon cancer cell line. (A) Quantification by FACS analysis of ROS species. H2DCF‐DA (2′,7′‐dichlorodihydrofluorescein diacetate) probe was used to quantify cytoplasmic hydrogen peroxide species; TMRM (tetramethylrhodamine methyl ester) to assess mitochondrial membrane potential (Ψm), and MitoSox‐Red probe for mitochondrial superoxide (SO ^·^) quantification. (B) Representative phase contrast pictures after treatment for 24 h with complete (25 mm glucose) or starved (0 mm glucose) media in the presence or absence of 5 mm/10 mm N‐acetyl‐cysteine (NAC). Scale bars: 100 μm. (C) Activation of survival pathways in DLD1‐miR661 colon cancer cell line. Total protein extracts were extracted after 24 h of treatment with complete media (25 mm glucose) or starved media (0 mm glucose) in the presence/absence of NAC (5 mm/10 mm). Western blot analysis of AMPK Thr172 phosphorylation and AMPK total protein. Levels of GSK3 phosphorylation detected by western blot using a phospho‐specific antibody (Ser21/9). Total GSK3β levels detection and β‐actin served as loading controls. Quantification of band intensity show the ratio of phosphorylated to total protein. *P < 0.05, **P < 0.01, ***P < 0.001.

To gain insights about the overall ROS status in DLD1‐miR661 cells, we analyzed the distribution of ROS species after treatment with low glucose or galactose (5 mm, 24 h) in the presence or absence of the antioxidant scavenger N‐acetylcysteine (NAC; 5 and 10 mm) (Fig. 2A). We did not find differences in cytoplasmic H_2_O_2_ levels in DLD1‐miR661 compared with DLD1‐Control cells, but mitochondrial superoxide anions (SO^·^) levels and mitochondrial membrane potential (Ψm) were higher in DLD1‐miR661 than in control cells. As expected, these differences increased after treatment with 5 mm galactose, a ‘ROS‐generating’ substrate as compared with 5 mm glucose. Importantly, NAC diminished the levels of SO^·^ in DLD1‐miR661 (galactose condition), although it did not change the mitochondrial membrane potential (Ψm) in DLD1‐miR661 cells. This indicates that miR661 increases SO^·^ at mitochondria, which can be partially alleviated with NAC; more importantly, miR661 seems to induce a metabolic reprogramming that favors the maintenance of high levels of mitochondrial membrane potential (Ψm) which is not rescued by NAC treatment. It is tempting to speculate that the latter may be a mechanism by which mitochondria is switched to an anabolic mode (discussed below).

We also checked cell sensibility to glucose deprivation. Glucose starvation inhibits the pentose phosphate pathway (PPP) which is required for NADPH production to detoxify ROS (Jeon *et al*., 2012). When glucose was diminished in the growing medium, DLD1‐miR661 cells became hypersensitive to glucose limitations, as seen by the reduction in the total number of cells. Importantly, treatment with the antioxidant NAC recovered the cell viability even at high doses (10 mm NAC) where the viability of DLD1‐Control cells was compromised (Fig. 2B).

It has been reported that cancer cells upregulate the KEAP1/NRF2 pathway to adapt to high levels of ROS and to the subsequent oxidative stress. The KEAP1/NRF2 pathway is a key pathway for sensing and responding to oxidative stress (Kansanen *et al*., 2013). To sustain Nrf2 at the nucleus, GSK3β inhibition by Akt‐mediated phosphorylation at Ser9 is required (Leinonen *et al*., 2014).

We checked this possibility in DLD1‐miR661 cells. As illustrated in Fig. 2C, DLD1‐miR661 cells displayed a significantly increased ratio of P‐(Ser9)GSk3β/TotalGSK3β compared with DLD1‐Control cells. Moreover, NAC treatment diminished this ratio to some extent, indicating that GSK3β inhibition seemed to be somehow dependent on ROS status.

As DLD1‐miR661 cells are sensitive to energy and oxidative stress, we also checked AMPK activation as a survival mechanism. As shown in Fig. 2C, the ratio of P‐(Thr172)AMPKα/TotalAMPKα) was significantly increased in DLD1‐miR661 cells compared with DLD1‐Control cells. AMPK, in addition to its function in ATP homeostasis, has a key role in NADPH maintenance (Lamouille *et al*., 2014). As glucose starvation inhibits the pentose phosphate pathway, cells require compensatory NADPH generation pathways. It has been proposed that the LKB1/CaMKK–AMPK/ACC1/2 axis has a key function in redox homeostasis by maintaining NADPH levels through fatty acid oxidation (FAO) induction (further discussed in metabolomic section 3.3) (Kottakis and Bardeesy, 2012). In addition, AMPK has been also described to turn off aerobic glycolysis (Warburg effect), favoring mitochondrial respiration. A critical mediator of this switch is pyruvate kinase M2 (PKM2). PK catalyzes the rate‐limiting ATP‐generating step of glycolysis in which phosphoenolpyruvate (PEP) is converted to pyruvate. PKM2 isoform provides an advantage to cancer cells because, by slowing glycolysis, it allows carbohydrate metabolites to be used for biosynthetic purposes (Christofk *et al*., 2008). Interestingly PKM2 protein was diminished in DLD1‐miR661 cells compared with control cells, although we cannot exclude an ROS‐mediated degradation at the protein level (Fig. 2C). We also confirm that N‐cadherin protein levels were higher in DLD1‐miR661 than DLD1‐Control cells (top panel) according to the miR661‐induced mesenchymal characteristics shown in Fig. 1. In summary, miR661 seems to activate survival pathways to manage energy and oxidative stress.

### 3.3 miR661‐induced metabolic profile supports its role as a regulator of redox and metabolic homeostasis

To determine the major pathways altered by miR661 overexpression in DLD1 CC cells, we performed a metabolomic analysis. Table 1A indicates significantly altered biochemicals with statistical significance (*P* ≤ 0.05) or with approaching significance (0.05 < *P* < 0.10) from a dataset analysis with a total 323 named biochemicals differentially detected in the study (red indicates significantly upregulated metabolites and green indicates downregulated ones) (File S1: total of significant metabolites ratio DLD1‐miR661/DLD1‐control (*P* < 0.05). The main metabolic pathways enriched upon miR661 overexpression are also shown (Table 1B). Briefly, a pathway enrichment value greater than one indicates that the pathway contains more experimentally regulated compounds relative to the study overall, suggesting that the pathway may be a target of interest of the experimental perturbation.

**Table 1:** Significantly altered biochemicals and pathway enrichment. (A) Biochemicals with statistical significance (*P* ≤ 0.05) or with approaching significance (0.05 < *P *< 0.10) from dataset analysis with a total 323 named biochemicals differentially detected in the study (red numbers indicate upregulated metabolites and green numbers, downregulated ones). Welch's two‐sample *t*‐test was used to identify biochemicals that differed significantly between experimental groups. (B) Pathway enrichment displays the number of experimentally regulated compounds relative to all detected compounds in a pathway, compared with the total number of experimentally regulated compounds relative to all detected compounds in the study. A pathway enrichment value greater than one indicates that the pathway contains more experimentally regulated compounds relative to the study overall, suggesting that the pathway may be a target of interest of the experimental perturbation (enrichment = number of significant metabolites in a specific pathway (*k*)/total number of detected metabolites in the specific pathway (*m*)/(total number of significant metabolites in all pathways (*n*)/total number of detected metabolites in the global analysis (*N*). The enrichment is calculated as [*k*/*m*)/(*n*/*N*)]

Significant altered biochemicals	Total biochemicals *P* < 0.05	Biochemical up/down	Total biochemicals 0.05 < *P* < 0.10	Biochemicals up/down
(A)				
miR661/control	294	98/196	29	14/15
(Welch's Two sample t‐test)				

Pathway name	Compounds in pathway			All compounds in study			Enrichment (*k*/*m*)/(*n*/*N*)
	Significant (*k*)	Detected (*m*)	Sig/Det (k/m)	Significant (*n*)	Detected (*N*)	Sig/Det (*n*/*N*)	
(B)							
Glycolysis, Gluconeogenesis, and pyruvate metabolism	11	11	1	294	453	0.65	1.54
Nucleotide sugar	6	6	1	294	453	0.65	1.54
Aminosugar metabolism	7	7	1	294	453	0.65	1.54
Glycogen metabolism	2	2	1	294	453	0.65	1.54
Pentose metabolism	7	9	0.78	294	453	0.65	1.2
Pentose phosphate pathway	4	5	0.8	294	453	0.65	1.23
Oxidative phosphorylation	2	2	1	294	453	0.65	1.54
TCA cycle	6	7	0.86	294	453	0.65	1.32
Creatine metabolism	3	4	0.75	294	453	0.65	1.16
Glutathione metabolism	7	8	0.88	294	453	0.65	1.35
Glutamate metabolism	6	9	0.67	294	453	0.65	1.03
Nicotinate and nicotinamide metabolism	8	9	0.89	294	453	0.65	1.37
Gamma‐glutamyl amino acid	7	11	0.64	294	453	0.65	0.98
Glycine, serine and threonine metabolism	5	6	0.83	294	453	0.65	1.28
Alanine and aspartate metabolism	3	5	0.6	294	453	0.65	0.92
Tryptophan metabolism	5	5	1	294	453	0.65	1.54
Histidine metabolism	6	7	0.86	294	453	0.65	1.32
Phenylalanine and tyrosine metabolism	5	8	0.62	294	453	0.65	0.96
Lysine metabolism	6	9	0.67	294	453	0.65	1.03
Urea cycle; Arginine and proline metabolism	8	12	0.67	294	453	0.65	1.03
Dipeptide	8	13	0.62	294	453	0.65	0.95
Pyrimidine metabolism, cytidine containing	4	7	0.57	294	453	0.65	0.88
Pyrimidine metabolism, uracil containing	6	10	0.6	294	453	0.65	0.92
Pyrimidine metabolism, Thymine containing	2	3	0.67	294	453	0.65	1.03
Purine and pyrimidine metabolism	1	1	1	294	453	0.65	1.54
Ketone bodies	1	1	1	294	453	0.65	1.54
Monoacylglycerol	11	13	0.85	294	453	0.65	1.3
Fatty acid, monohydroxy	2	3	0.67	294	453	0.65	1.03
Fatty acid, dicarboxylate	3	3	1	294	453	0.65	1.54
Carnitine metabolism	2	2	1	294	453	0.65	1.54
Fatty acid metabolism (also BCAA metabolism)	2	2	1	294	453	0.65	1.54
Fatty acid synthesis	2	2	1	294	453	0.65	1.54
Sterol	2	3	0.67	294	453	0.65	1.03
Phospholipid metabolism	19	33	0.58	294	453	0.65	0.89
Sphingolipid metabolism	5	6	0.83	294	453	0.65	1.28

Table 2 summarizes main metabolites altered, grouped by pathways.

**Table 2:** Summary of significantly metabolites altered by miR661 overexpression in colon cancer cells grouped in pathways. Fold change miR661/control of significant changed metabolites

Sub pathway	Biochemical name	miRNA Ctrl+Scrm
Glycolysis, gluconeogenesis, and pyruvate metabolism	Glucose	0.01
	Glucose‐6‐phosphate (G6P)	0.15
	Glucose 1‐phosphate	0.12
	Fructose‐6‐phosphate	0.26
	Dihydroxyacetone phosphate (DHAP)	2.70
	3‐phosphoglycerate	0.23
	Phosphoenolpyruvate (PEP)	0.13
	Pyruvate	2.51
	Lactate	0.71
	Glycerate	0.12
Pentose phosphate pathway	6‐phosphogluconate	0.01
	Ribose 1‐phosphate	1.43
	5‐phosphoribosyl diphosphate (PRPP)	0.02
	Sedoheptulose‐7‐phosphate	0.56
	Ribulose/xylulose 5‐phosphate	0.14
Pentose metabolism	Ribulose	0.23
	Ribose	0.14
	Ribitol	6.58
	Ribonate	0.30
	Xylonate	0.35
	Xylitol	0.12
	Arabitol	1.44
Glycogen metabolism	Maltotetraose	0.05
	Maltotriose	0.22
Fructose, mannose and galactose metabolism	Fructose	0.21
Nucleotide sugar	UDP‐glucose	0.10
	UDP‐galactose	0.29
	UDP‐glucuronate	0.09
	UDP‐IM‐acetylglucosamine	0.27
Aminosugar metabolism	Glucosamine‐6‐phosphate	0.20
	Glucuronate	0.54
	*N*‐acetylglucosamine	0.49
	*N*‐acetylglucosamine 6‐phosphate	0.08
	*N*‐acetyl‐glucosamine 1‐phosphate	0.10
TCA cycle	Citrate	1.82
	Succinylcarnitine	0.36
	Succinate	0.38
	Fumarate	0.83
	Malate	0.83
	2‐methylcitrate/homocitrate	3.11
Glutamate metabolism	Glutamate	0.68
	*N*‐acetylglutamate	8.38
	*N*‐acetylglutamine	3.84
	*N*‐acetyl‐aspartyl‐glutamate (NAAG)	67.89
	Gamma‐aminobutyrate (GABA)	1.31
	4‐methylglutamate	1.95
Urea cycle; Arginine and proline metabolism	Arginine	0.52
	Proline	0.51
	Citrulline	0.49
	Argininosuccinate	0.50
	Homocitrulline	0.23
	Dimethylarginine (SDMA + ADMA)	2.50
	*N*‐acetylarginine	0.61
	*N*‐delta‐acetylornithine	0.39
	Trans‐4‐hydroxyproline	0.42
Creatine metabolism	Creatine	2.49
	Creatinine	1.40
	Creatine phosphate	0.09
	Guanidinoacetate	0.57
Glutathione metabolism	Glutathione, reduced (GSH)	2.23
	Glutathione, oxidized (GSSG)	6.99
	Cysteine‐glutathione disulfide	1.41
	S‐methylglutathione	2.93
	Cysteinylglycine	0.44
	S‐nitrosoglutathione (GSNO)	4.44
aa	Down	
N‐acetylated aa	Up	
Gamma‐glutamyl aa	Down	
Dipeptides	Down	
Fatty acid synthesis	Malonylcarnitine	0.19
	2‐Methylmalonyl carnitine	0.09
Fatty acid metabolism	Acetyl CoA	2.22
Fatty acid metabolism (BCAA Metabolism)	Butyrylcarnitine	1.96
	Propionylcarnitine	2.18
Fatty acid metabolism (Acyl Carnitine)	Valerylcarnitine	2.37
	Hexanoylcarnitine	1.68
	Palmitoylcarnirine	2.71
Carnitine metabolism	Deoxycarnitine	0.58
	Carnitine	0.52
Ketone bodies	3‐Hydroxybutyrate (BHBA)	1.84
Glycerolipid metabolism	Glycerol 3‐phosphate (G3P)	0.11
Monoacylglycerol	1‐Myristoylglycerol (14:0)	3.12
	2‐Myristoylglycerol (14:0)	4.18
	1‐Palmitoylglycerol (1‐monopalmitin)	2.85
	2‐Palmitoylglycerol (16:0)	4.84
	1‐Margaroylglycerol (17:0)	3.03
	1‐Oleoylglycerol (18:1)	3.65
	2‐Oleoylglycerol (18:1)	4.95
	2‐Linoleoylglycerol (18:2)	4.09
	1‐Docosahexaenoylglycerol (22:6)	2.32
	2‐Docosahexaenoylglcyerol^a^	4.65
	2‐Palmitoleoylglycerol (16:1)^a^	1.69

a Indicates compounds that have not been officially confirmed based on a standard, but we are confident in its identity.

Glycolysis and pyruvate metabolism were profoundly altered by miR661. Glycolytic and pentose phosphate pathway‐related metabolites were strikingly downregulated (nucleotide sugars, pentoses and aminosugars), except dihydroxyacetone phosphate (DHAP) and pyruvate (Pyr). In general, aminoacids were diminished, but not the corresponding acetylated aminoacids. Importantly, glutathione metabolism was upregulated. Moreover, glutamine was upregulated but not glutamate. This seems to indicate an increase in glutamine uptake and quick utilization of glutamate for TCA cycle anaplerosis, and for the synthesis of glutathione (Table 2, File S1).

In addition, fatty acid (FA) biosynthesis and cholesterol were diminished, but intermediates of FA mobilization (monoacylglycerols, ketone bodies and AcCoA) presented the opposite tendency. All the TCA intermediates except citrate were downregulated. To summarize, miR661 induces a clear depletion of anabolic pathways from glycolytic intermediates and TCA cycle, and displays a higher dependency on FAO intermediates (Table 1B, File S1).

### 3.4 miR661 compromises glycolysis and mitochondrial respiration

To gain insights into how these metabolomic differences translated into functional bioenergetics, we monitored, by flux analysis, extracellular acidification (ECAR) and oxygen consumption rates (OCR).

**Figure 3:**
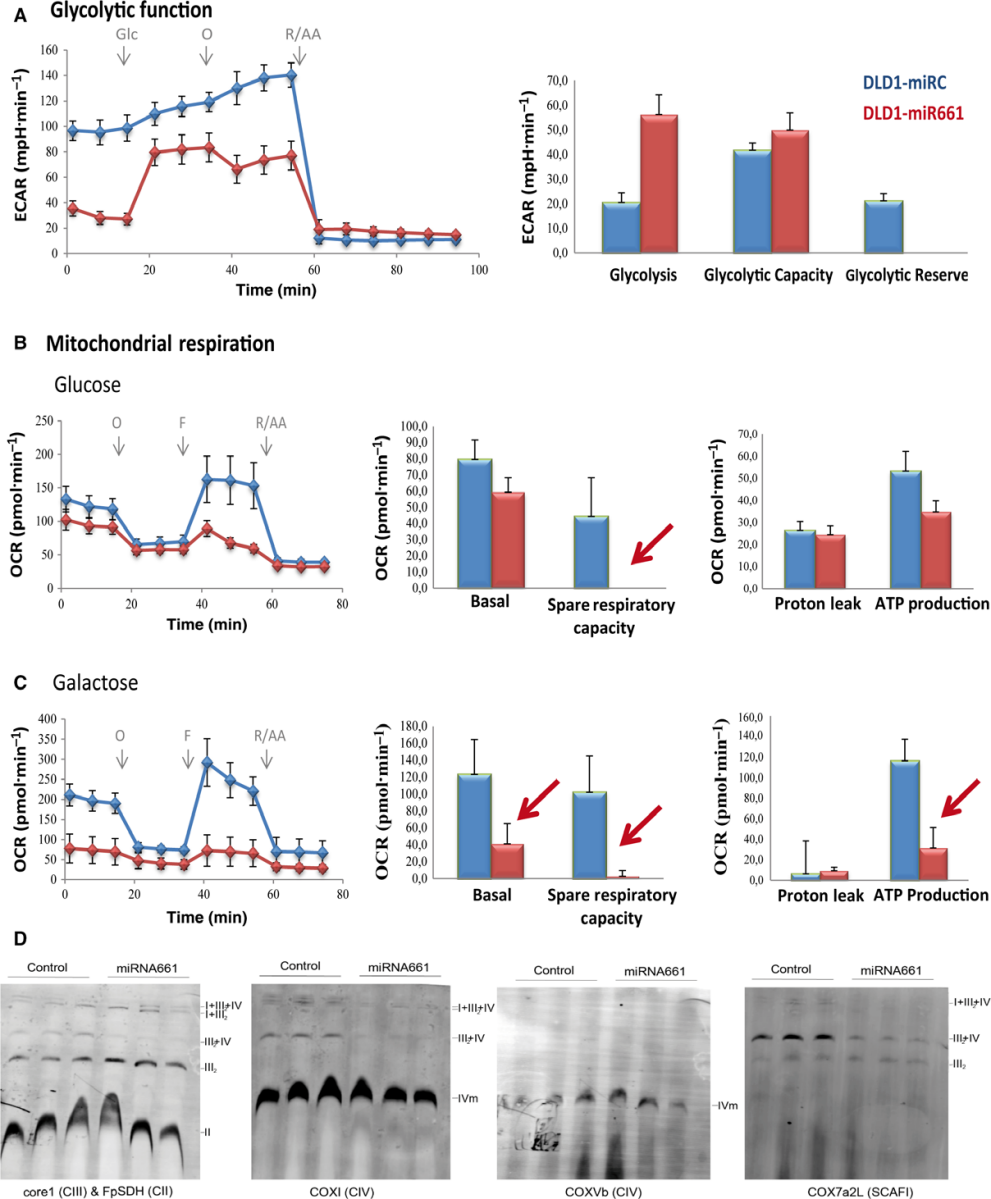
MicroR661 compromises both glycolytic function and mitochondrial respiration in DLD1 colon cancer cells. Comparison of the glycolytic function and mitochondrial function of DLD1‐miR661 vs. control cells: Glycostress assay and Mitostress standard assays. (A) Glycostress: cells were maintained in 5 mm glucose overnight (o/n). The following day, cells were glucose‐starved for 1 h and basal ECAR was monitored. Upregulation of ECAR after 10 mm glucose injection was used to determine glycolysis. Maximal ECAR (stressed glycolysis) was determined after oligomycin injection. Mitochondrial respiration analysis in the presence of 10 mm glucose (B) and 10 mm galactose (C) with standard MitoStress assay. Basal OCR, OCR dedicated to ATP production, maximal respiration rate (MRR) and H^+^ leak are shown. (D) Immunoblot of assembled supercomplexes (SCs) in digitonin‐permeabilized mitochondria separated by BNGE and probed with monoclonal antibodies for CIII (anti‐Core1), CIV (anti‐CoxI) (anti‐CoxVb) and CII (FpSDH). Immunoblot probed with ScafI (Cox7a2L) shows that III_2_ IV_1_ SCs were reduced in DLD1‐miR661. The amounts of CIII_2_ were slightly increased but III_2_‐IV_1_ SCs were reduced in DLD1‐miR661 cells compared with control cells.

Basal ECAR of miR661 cells (three measurements before oligomycin injection) was dramatically affected compared with control cells, in line with the intracellular depletion of glucose and glycolytic intermediates observed by metabolomic analysis (Table 2, File S1). After the injection of glucose, DLD1‐miR661 cells upregulated glycolysis from the basal situation, to a higher extent compared with control cells (Fig. 3A). This indicates that DLD1‐miR661 cells do not rely on aerobic glycolysis in a basal situation; however, when challenged to use exogenously added glucose after starvation, they have capacity to undergo glycolysis. When ATPase was inhibited with oligomycin to block ATP production from mitochondria, DLD1‐miR661 cells were not able to increase glycolysis, indicating an altered mitochondrial respiration as oligomycin injection, does not contribute to upregulate ECAR. Since ECAR measurement is an indirect read‐out of l‐lactate production from glycolysis, we analyzed l‐lactate levels in the media. Proliferating DLD1‐miR661 cells (50% confluency) had reduced l‐lactate levels compared with the DLD1‐parental cell line (Fig. S2).

Next, we monitored OCR in response to well defined modulators of mitochondrial function. DLD1‐miR661 cells displayed a reduced basal respiration rate (BRR) compared with control cells (three measurements before oligomycin injection). When oligomycin (1 μm) was injected to inhibit ATPase, OCR decreased to similar levels (four to six measurements) as control cells. However, when FCCP (0.4 μm) was used to free the H^+^ gradient through the inner mitochondrial membrane and uncouple the electron transporter system (ETC) from the synthesis of ATP, the maximal respiration rate (MRR) was strongly reduced in DLD1‐miR661 cells (Fig. 3B). MMR, which is a readout of the maximal capacity of respiration at mitochondria and an indirect measurement of mitochondrial fitness, was clearly compromised in DLD1‐miR661 cells. This agrees with the increased levels of mitochondrial membrane potential (Ψm), although additional effects related to mitochondrial cell damage may also be implicated.

We also performed a mitoStress test in the presence of 10 mm galactose, a stronger ROS‐generating substrate. As expected, galactose‐treated DLD1‐Control cells raised basal OCR level compared with the glucose condition. In contrast, a dramatic decrease in the basal OCR for DLD1‐miR661 was found. This might be a consequence of the extreme membrane potential that DLD1‐miR661 cells face. Moreover, DLD1‐miR661 cells displayed similar levels of both MRR and BRR, which is an indication that DLD1‐miR661 cells are at their top limit of respiration and therefore have a reduced mitochondrial function (Fig. 3C). Depending on their genetic background and mutational status, cancer cells rely on glycolysis and/or mitochondrial respiration. However, miR661 seems dramatically to compromise both functions in DLD1 cells. These data suggest that miR661 plays an important role in regulation of energy and redox homeostasis in DLD1 human colon cancer cells.

To obtain an insight into how DLD1‐miR661 cells manage mitochondrial respiration in the presence of high ROS, we studied the assembly of mitochondrial complexes into supercomplexes (SCs). The analysis of the SC assemblage reports the electron flux through complexes of the mitochondrial electron transport chain (ETC). Blue‐native gel electrophoresis (BNGE) showed that DLD1‐miR661 had decreased levels of CIII associated in CI‐CIII‐CIV SC and increased levels of free‐CIII complex and CIII‐CIV SC (Fig. 3D). On one hand, the decreased levels of CI‐CIII‐CIV SC in DLD1‐miR661 indicate that these cells switch off respiration through CI to favor respiration through CII and/or directly to CoQ. By doing this, DLD1‐miR661 cells may preserve the limited pool of NAD^+^ and NADH (File S1). This is also in agreement with preferential mobilization of FAs (File S1) vs. other substrates to fuel mitochondrial respiration (possibly via the electron‐transfering flavoprotein). In addition, as revealed by the analysis of the SCAFI adaptor protein, it was found that DLD1‐miR661 cells favor free CIII over CIII‐CIV assembly (Fig. 3D, right panel). By doing so, DLD1‐miR661 cells maintain high levels of membrane potential, which also contributes to the use of TCA intermediates for biosynthesis instead of oxidative phosphorylation. This is in agreement with the decreased levels of BRR and MRR that reflect a reduced catabolism to preserve anabolism, a mechanism of adaptation already described for LONP1‐overexpressing cells (Quirós *et al*., 2014). In summary, these results reflect a miR661‐dependent metabolic reprogramming affecting both glycolysis and mitochondrial respiration to preserve anabolic processes (mitochondrial anabolic mode).

### 3.5 miR661 overexpression in metastatic CC cells alters functional cell bioenergetics

Epithelial to mesenchymal transition‐activating ROS can result from different processes such as a general oxidative stress, activation of ROS‐producing enzymes, or stromal cells in the tumor microenvironment. Moreover, the specificity of action of ROS for induction of EMT is not absolute, and is strictly dependent upon tissue type and cellular context. To further segregate the EMT‐associated effects on cell metabolism from other miR661 metabolic targets, we overexpressed miR661 in the invasive SW620 colon cancer cell line (Fig. S3A). The SW620 cell line does not present a mesenchymal phenotype (N‐cadherin) but, instead expresses *Cytokeratin 18* and *Vimentin* (Hewitt *et al*., 2000). As shown in Fig. S3B, unlike DLD1 cells, both SW620‐control and SW620‐miR661 expressed high levels of *Vimentin*. Moreover, the expression levels of *Snail, Slug* and *Vimentin* were not affected by miR661 overexpression in SW620 cells compared with control cells (*Nectin* expression was used as a validated described target for miR661) (Fig. S3C). In addition, we monitored invasion through BD‐coated Matrigel Chambers and found only a slight, not significant, decrease in invasiveness of SW620‐miR661 compared with SW620‐Control (Fig. S3D). It seems that miR661 does not alter the expression of EMT markers or the invasiveness capability of SW620 metastatic cancer cell line.

**Figure 4:**
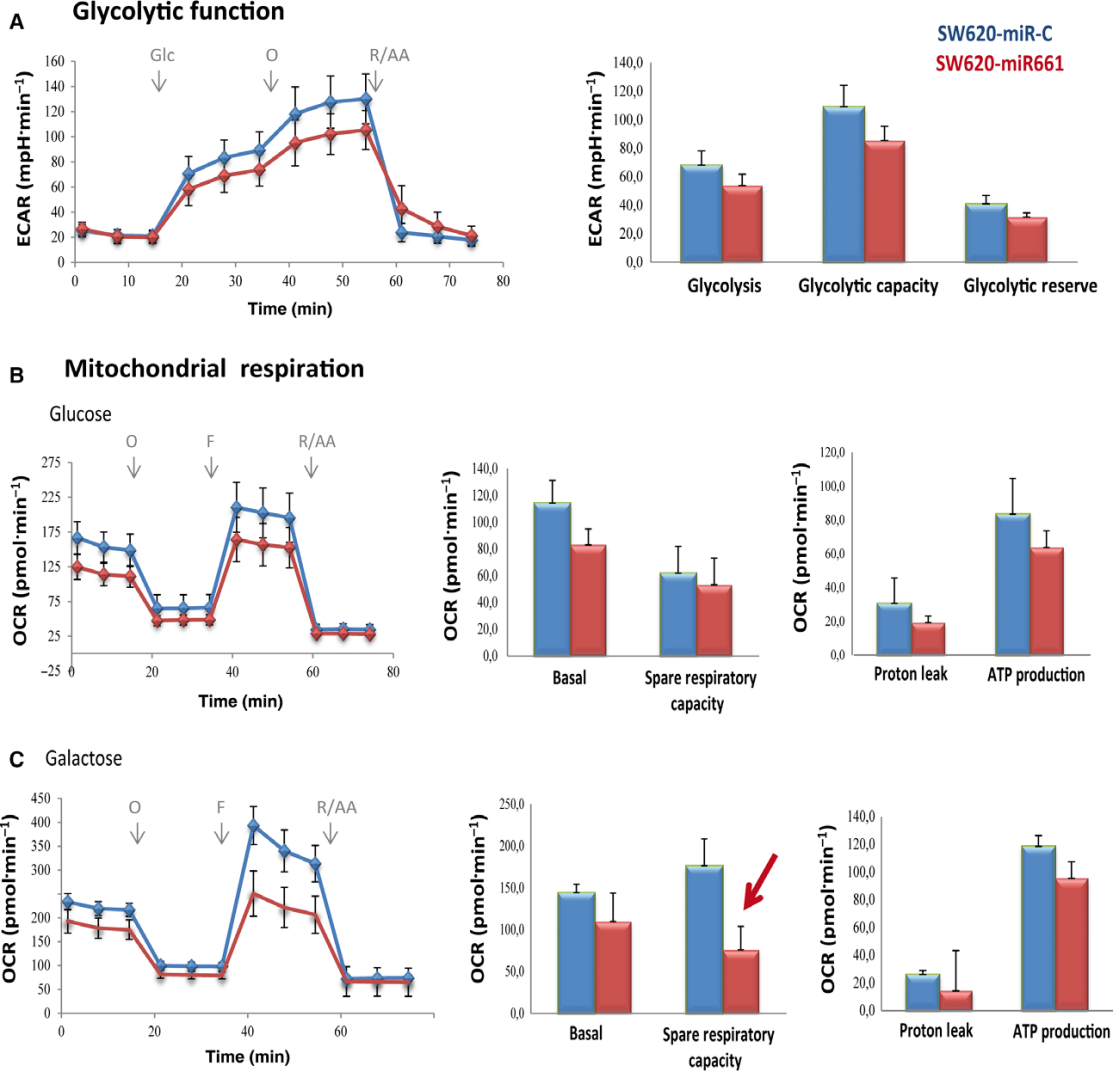
MicroR661 compromises glycolysis and mitochondrial respiration in SW620 metastatic colon cancer cell line. (A) Analysis of ECAR glycolysis stress test. Cells were maintained in 5 mm glucose overnight (o/n). The following day, cells were glucose‐starved for 1 h and basal ECAR was monitored. Upregulation of ECAR after 10 mm glucose injection, was used to determined glycolysis. Maximal ECAR (stressed glycolysis) was determined after oligomycin injection. Analysis of OCR mitostress test in glucose (B) or galactose (C). Basal OCR was monitored in complete media. OCR dedicated to ATP production was determined after oligomycin injection. Uncoupling ETC from ATP production after FCCP injection was used to determine MRR.

Quantitative analysis of metabolism of CC cell lines have revealed that their reliance on glycolysis and/or mitochondrial respiration is cell line‐dependent, which might be a consequence of the mutational status of the cancer cells (Zaytseva *et al*., 2015). Thus, we wanted to address the effect of miR661 in SW620 metastatic CC cell line. Interestingly, basal ECAR of SW620‐Control cells was strikingly lower than that of the DLD1‐Control cells, indicating that the SW620 cell line seems to be less dependent on glycolysis compared with DLD1 cells (Fig. 4A). We observed a slight decrease in the maximal glycolytic capacity in SW620‐miR661 compared with control cells, although this was not significant. Next, we monitored mitochondrial response under stressed conditions by running a mitoStress standard assay (Fig. 4B). Significant differences were found in the spare respiratory capacity and, more specifically, in the presence of ‘ROSgenic’ galactose as substrate (Fig. 4C, red arrow), again pointing towards a role of miR661 in increasing the overall oxidative stress.

### 3.6 PKLR and H6PDH are diminished in miR661 overexpressing CC cells

**Figure 5:**
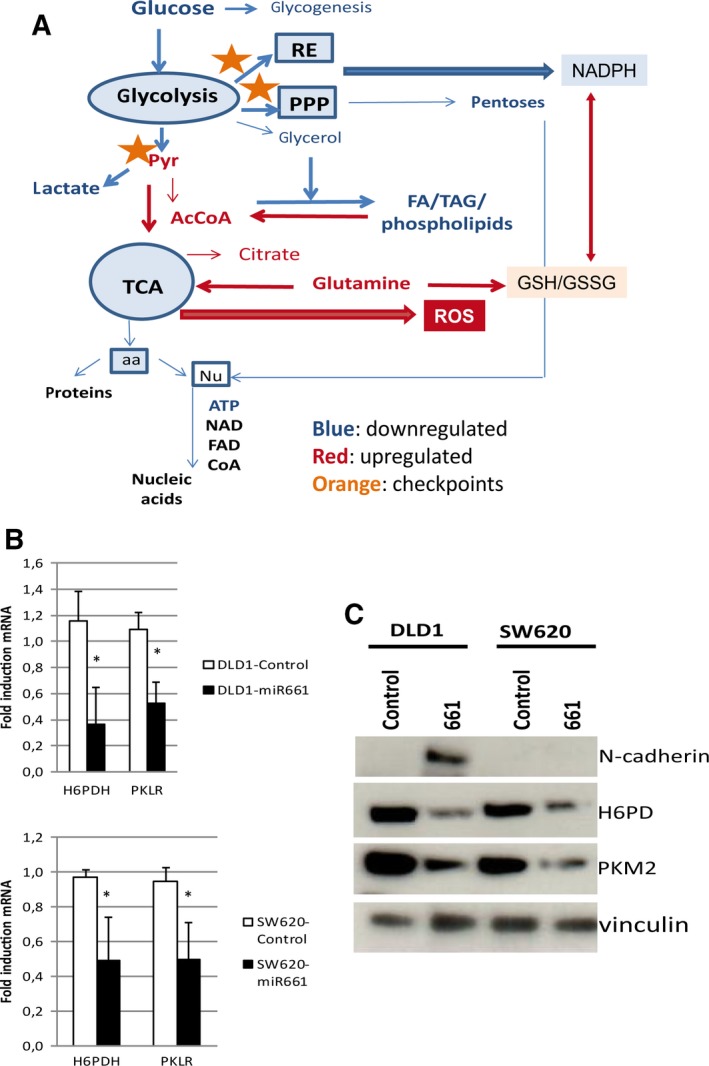
Pathways and targets modulated by miR661 overexpression in colon cancer DLD1 cell line. (A) Proposed model of the main altered pathways as well as key related enzymes (orange asterisks). *In silico* bioinformatic prediction of miR661 targets related to cell metabolism, indicated that PKLR and H6PD were the two main candidates. (B) qRT‐PCR analysis of *PKLR* and *H6PDH* levels as predicted targets in DLD1 and SW620 overexpressing miR661 compared with the corresponding controls. (C) WB for both H6PD and PKM2 in DLD1‐Control and DLD1‐miR661, and SW620‐Control and SW620‐miR661. aa, aminoacids; FA, fatty acids; Nu, nucleotides; PPP, pentose phosphate pathway; RE, endoplasmic reticulum; TAG: triacylglycerols; TCA, tricarboxylic acid.

Metabolomic data and functional bioenergetics analysis were combined to gain insight into the molecular mechanisms involved in the regulatory role of miR661 on oxidative stress and metabolic reprogramming. Figure 5A shows the proposed model for the main pathways that were altered, as well as key related enzymes (asterisks).

*In silico* bioinformatic prediction of miR661 targets related to cell metabolism pointed to *PKLR* and *H6PD* as two main candidates. Overexpression of miR661, both in DLD1 and SW620 cells, diminished the expression levels of *PKLR* and *H6PD* (Fig. 5B). Pyruvate kinase liver red blood enzyme (PKLR) is implicated in the conversion of PEP to pyruvate, specifically in liver and red blood cells. We therefore hypothesized that miR661 may also target the M isoform, which has been described to be highly expressed in cancer cells (Mazurek, 2011). PKM2 increases the diversion of glycolytic intermediates into anabolic pathways, including the pentose phosphate pathway and also slows down ATP generation (Anastasiou *et al*., 2011). In addition, PKM2 translocation into the nucleus has been described, where it functions as a histone kinase upregulating the expression of c‐Myc and cyclin D1, thereby promoting the Warburg effect and cell cycle progression, respectively (Yang and Lu, 2013). Hexose‐6‐phosphate dehydrogenase (H6PDH), similar to glucose‐6‐phosphate dehydrogenase (G6PD), is important for NADPH production and for the global redox homeostasis (Lavery *et al*., 2006). H6PDH is specifically responsible for NADPH generation at the endoplasmic reticulum (ER) lumen, which is also essential for the proper function of ER (Szaraz *et al*., 2010). As shown in Fig. 5C, both H6PDH and PKM2 protein levels were diminished in DLD1‐ and SW620 miR661‐overexpressing cells.

By *in silico* prediction, the *H6PD*‐3′UTR region contains at least 24 putative binding sites for miR661, and *PKM*‐3′UTR at least five. Based on mirSVR scores (not shown), we selected short regions (500–600 bp) of the 3′UTR sequences of *H6PD* and *PKM*, containing the binding sites with the highest mirSVR scores, to perform luciferase assays. Luciferase analyses showed no differences among constructs containing the wt‐predicted binding sites and the mutated ones (Fig. S4). Although we were not able to provide a mechanistic demonstration that miR661 directly regulates H6PD and PKM, the complete 3′UTR sequences contain additional predicted binding sites for miR661 that have not been analyzed here. In addition, it is possible that miR661 interactions may need secondary structures only achieved with the complete 3′UTR sequences, and/or the observed differences in the mRNA levels of H6PD and PKM after miR661 overexpression may occur by indirect mechanisms. Nevertheless, although not demonstrated by direct interaction, the final read‐out of miR661 overexpression in both cell lines (DLD1 and SW620) is the depletion of H6PDH and PKM at the transcriptional (qRT‐PCR, Fig. 5B) and post‐transcriptional levels (WB, Fig. 5C). Moreover, it cannot be discarded that PKM2 protein depletion may also be the result of a combination of the effect of miR661 at the post‐transcriptional level and/or an indirect effect of the oxidative stress on protein stability.

### 3.7 Reexpression of H6PDH and PKM2 in DLD1‐miR661 leads to a partial rescue of cell bioenergetics

**Figure 6:**
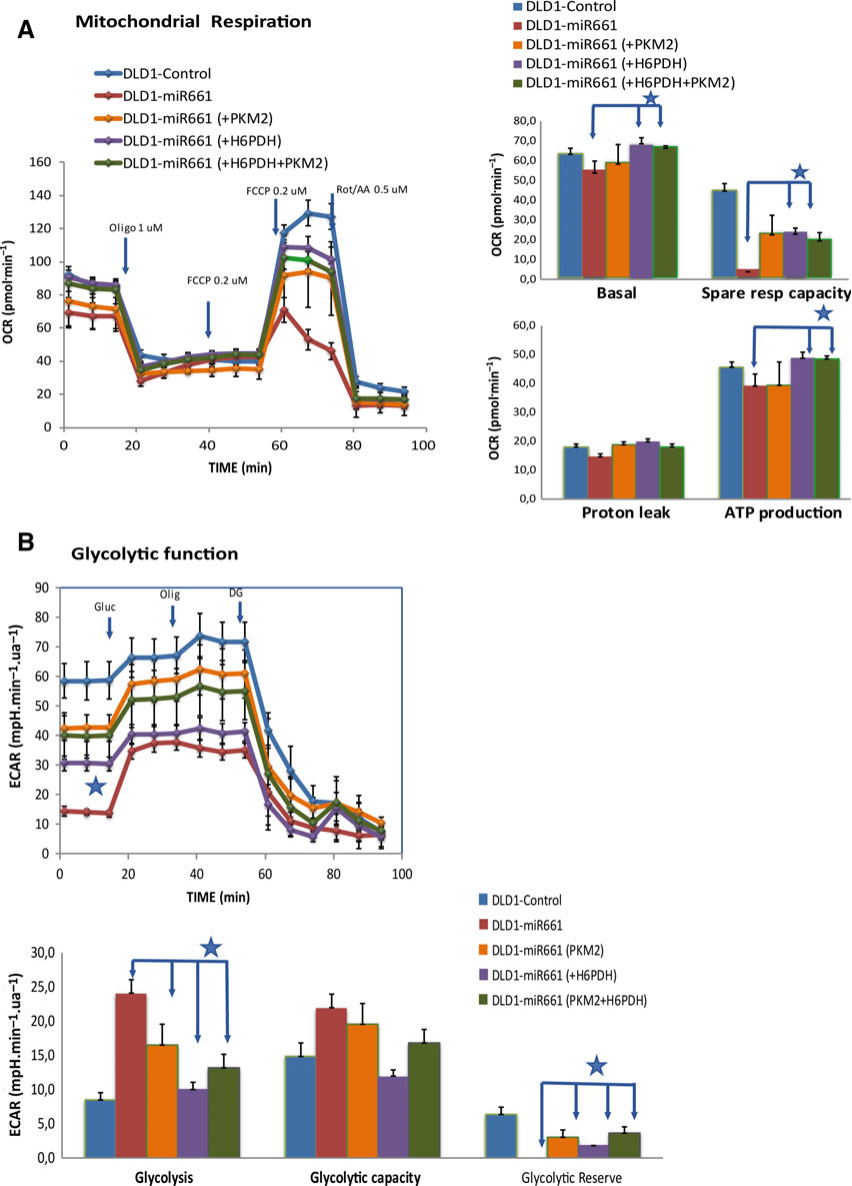
Re‐expression of H6PDH and PKM2 in DLD1‐miR661 leads to a partial rescue of cell bioenergetics. MitoStress and GlycoStress standard assays comparison of DLD1‐miR661‐H6PDH, DLD1‐miR661‐PKM2 and DLD1‐miR661‐H6PD‐PKM2 with DLD1‐miR661 and DLD1‐Control cells. (A) Mitochondrial respiration analysis in the presence of 10 mm glucose. Responses in OCR after oligomycin injection (1 μm), FCCP injection (two sequential additive injections of 0.2 μm) and Rotenone/Antimycin (0.5 μm). (B) Basal ECAR (after 1 h of glucose starvation) and responses after 10 mm glucose, 1 μm oligomycin and 50 mm DG injections. *P < 0.05, **P < 0.01, ***P < 0.001.

To demonstrate that miR661‐associated downregulation of H6PDH and PKM2 had a direct effect on the observed changes in cell metabolism, we transiently overexpressed H6PDH (DLD1‐miR661‐H6PD), PKM2 (DLD1‐miR661‐PKM2) and both targets simultaneously (DLD1‐miR661‐H6PD‐PKM2) in DLD1‐miR661 cells. Figure 6 shows Mitostress and Glycostress standard assays with DLD1‐Control, DLD‐miR661, DLD1‐miR661‐H6PD, DLD1‐miR661‐PKM2 and DLD1‐miR661‐H6PD‐PKM2 cells. Regarding mitochondrial function, we found a partial rescue of basal OCR and spare respiratory capacities in DLD1‐miR661‐H6PD and DLD1‐miR661‐H6PD‐PKM2 compared with DLD1‐Control and DLD1‐miR661 situations. The effect on these parameters was less prominent in DLD1‐miR661‐PKM2. Regarding glycolytic function, DLD1‐miR661‐PKM2 and DLD1‐miR661‐H6PD‐PKM2 upregulated basal ECAR (basal glycolysis after 1 h of glucose starvation) as well as the glycolytic reserve compared with DLD1‐miR661. In contrast, glycolysis (response to glucose after starvation) was reduced, becoming closer to the DLD1‐Control situation. These effects were less marked in DLD1‐miR661‐H6PD cells. It is tempting to speculate that H6PD has a direct effect on the mitochondrial oxidative respiration, and that PKM2 contributes directly to glycolysis.

### 3.8 Expression levels of miR661 in colon cancer patients

**Figure 7:**
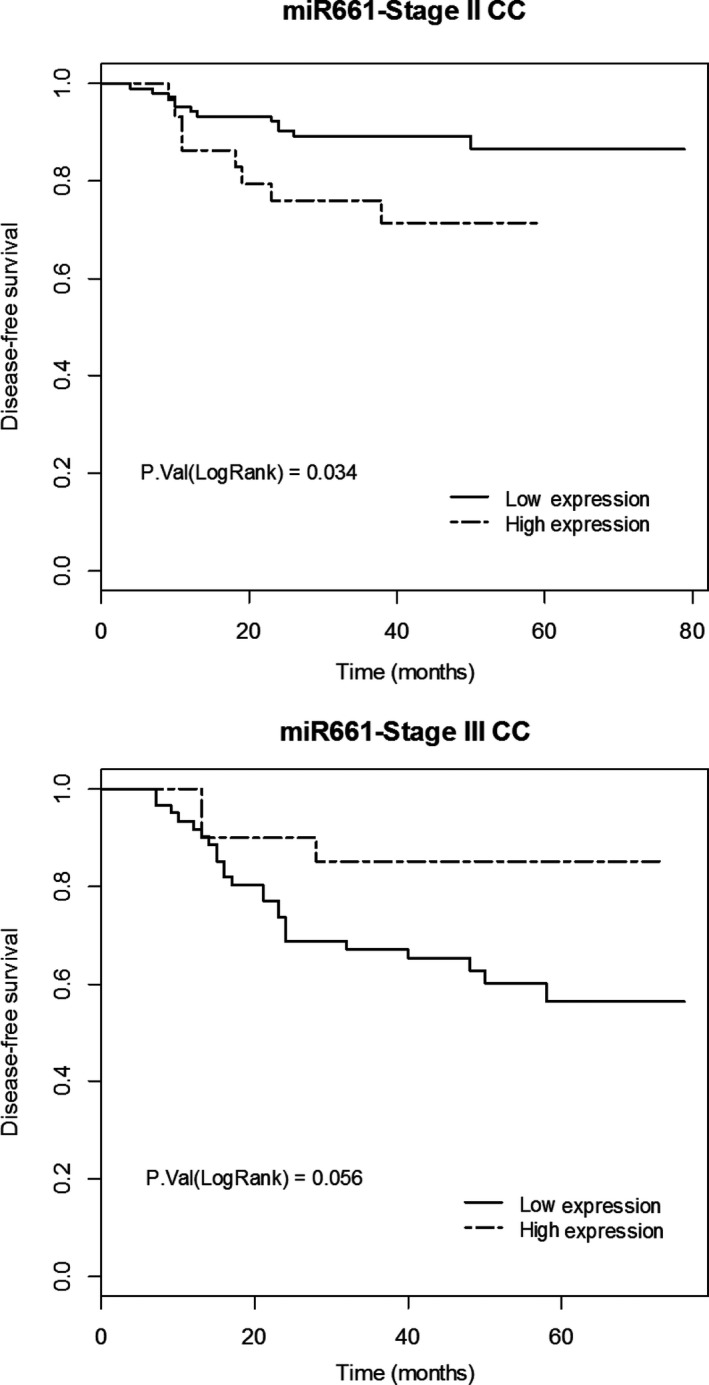
MicroR661 association with clinical prognosis of stage‐II and stage‐III CC patients. Kaplan–Meier plots for disease‐free survival showed an association between expression levels of miR661 and clinical outcome in stage‐II CC patients (*n *= 136; 77.2% low miR661 expression with 11.4% relapse vs. 22.8% high expression with 25.8% relapse) and stage‐III CC patients (*n *= 81; 75.3% low miR661 expression with 39.3% relapse vs. 24.7% high expression with 15% relapse).

To investigate the clinical relevance of miR661 in CC we analyzed the putative association between the expression levels of miR661 and the clinical outcome in tumor samples from stage II (*n *= 136) and stage III (*n *= 81) CC patients (Table S1). Kaplan–Meier plots for disease free‐survival showed an association between high expression levels of miR661 and poorer clinical outcome in stage‐II CC patients. But importantly, high miR661 expression levels in stage‐III CC patients tends to correlate with better, although not significant, prognosis (Fig. 7). Although these data need to be further validated in independent cohorts of stage‐II and stage‐III CC patients, it is tempting to hypothesize that the effect of miR661 in DLD1 non‐metastatic cells might mimick the situation in stage‐II CC, and the effect on SW620 may mimick the more advanced stage‐III CC. Hence, miR661 seems to induce an oxidative stress which promotes EMT in the non‐metastatic DLD1 cells, correlating with the poorer prognosis in stage‐II CC patients. However, in a more advanced stage, similar to results in SW620 metastatic cells, increased miR661 levels might raise the oxidative stress to a threshold that is incompatible with cell survival.

## 4 Discussion

Tumor cells reprogram their metabolism to support rapid and uncontrolled growth. The ability to manage and overcome the associated metabolic stress is crucial for cancer cell survival and metastasis (Jeon *et al*., 2012). It is well known that oncogenic transformation alters cellular metabolism to facilitate cell proliferation, but less is known about the metabolic changes that promote EMT and metastasis. ROS have been extensively associated to EMT by the promotion of oncogenic mutations (Gupta *et al*., 2012) and/or activation of EMT‐related signaling pathways (Cichon and Radisky, 2014). ROS can be generated by different mechanisms such as exposure to matrix metalloproteinases (MMPs; Radisky *et al*., 2005), inflammation (Coussens and Werb, 2002), and as a result of byproducts of cell metabolism. In this sense, a role of carbohydrates in the induction of EMT has been described (Lin *et al*., 2012), and we recently have reported a role of lipid metabolism‐related genes in the induction of EMT in colon cancer cell lines (Sanchez‐Martinez *et al*., 2015).

Herein we describe a role for microRNA‐661 as an epigenetic regulator of CC cell metabolism. MicroR661 induces a global increase in ROS, specifically SO^‐^ derived from mitochondria, that confers an EMT phenotype to non‐metastatic colon cancer cells but at the same time makes them critically sensitive to oxidative stress. This effect seems to be a general effect of miR661 in colon cancer, as overexpression of miR661 in the metastatic SW620 colon cancer cell line diminishes its tolerance to metabolic stress without altering its invasiveness properties. We hypothesized that miR661 might target metabolic genes, explaining its biological function and characterizing the role of miR661 in cell metabolism, integrating data from metabolomic analysis with functional cell bioenergetics. We found a diminished carbon flux through aerobic glycolysis (reduced l‐lactate production and altered glycolytic function) upon miR661 overexpression. Metabolomic data indicated that both branches of the pentose phosphate pathway (PPP) were diminished (File S1: (i) reduced levels of nucleotide sugars and aminosugars; (ii) decreased pentose phosphates intermediates such as 5‐phosphoribosyl diphosphate (PRPP), which suggests that nucleoside phosphorolysis preserves pentose phosphates). In addition, upregulation of glutamate and glutathione metabolism together with the increase of N‐acetylated aminoacids at the expense of the corresponding aminoacids indicates a metabolic adaptation to maintain redox homeostasis (Table 2, File S1). In addition, we observed an increase on AMPK activation that may activate FA mobilization for FAO. Several features support this idea. First, metabolomic data indicated increased levels of intermediates of FA mobilization (monoacylglycerols, ketone bodies and AcCoA). Secondly, the analysis of the reorganization of SC at the mitochondrial ETC (reduced CI‐CIII‐CIV SC, but increased CIII‐CIV and free CIII) suggests a metabolic adaptation to fuel ETC directly to CIII‐CIV and CIII from NADH/FADH_2_ produced from FAO. This reorganization allows TCA intermediates (mainly malate and citrate) to be used to regenerate cytoplasmic NADPH via malic enzyme (ME) and isocitrate dehydrogenase (IDH1), respectively (Hardie and Pan, 2002). Data from functional cell bioenergetic analysis indicated that overexpression of miR661 diminished the glycolytic function in the glycolytic cell line DLD1. This effect was less prominent in the SW620 cell line, which has been described as less dependent on glycolysis. Importantly, mitochondrial respiration was affected in both cell lines, possibly due to the disruption in redox homeostasis. To summarize, miR661 induces profound changes on cell metabolism: diminished aerobic glycolysis, downregulation of pentose phosphate metabolism, and breakdown of redox homeostasis.

*In silico* prediction of miR661 targets related to cell metabolism pointed to *PKLR* and *H6PD* as the two main candidates, which was validated by gene expression analysis (Fig. 5B). Regardless of PK, we hypothesized that miR661 may also target the M2 isoform, which is frequently overexpressed in cancer cells, similar to embryonic tissues. PKM2 increases the diversion of glycolytic intermediates into anabolic pathways, including the pentose phosphate pathway which contributes to the NADP^+^/NADPH balance and redox homeostasis. In addition, PKM2 translocation into the nucleus leads to the upregulation of c‐Myc and cyclin D1, promoting the Warburg effect and cell cycle progression, respectively. Regardless of H6PD, this is crucial for NADPH production and for the global redox homeostasis (Lavery *et al*., 2006; Szaraz *et al*., 2010). It has been reported that H6PD knockdown induces autophagy as a survival mechanism up to a threshold of oxidative injury, after which cells undergo apoptosis (Kapuy and Banhegyi, 2013). Although we have not demonstrated a direct effect of miR661 in PKM and H6PD downregulation by luciferase assays using short regions of their 3′UTRs, our results showed that miR661 downregulated mRNA and protein levels of PKM2 and H6PD in non‐metastatic and metastatic CC cell lines.

Finally, the preliminary analysis of miR661 status in CC patients indicated an association between high expression levels of miR661 and poorer clinical outcome in early‐stage CC patients, contrasting with the effects observed for more advanced stage‐III patients, in agreement with *in vitro* data with non metastatic and metastatic CC cells. Based on all these findings, we suggest that miR661 is a potential relevant epigenetic regulator of redox homeostasis and cell metabolism in CC.

## Data accessibility

**File S1**. Significantly altered biochemicals. Data were normalized by protein concentration, and Heat Map represents statistically significant biochemicals profiled in the analysis. Ratios between mean values in DLD1‐ miR661 and DLD1‐Control for each metabolite are indicated. Green colour indicates fold change ≤ 1 with a *p* < 0.05, and red colour indicates a fold change ≥1 with a *p* < 0.05.

## Author contributions

MG‐C and AR‐M conceived and designed the project. Experimental procedures were conducted by MG‐C, RS‐M, RA and SM. The manuscript was prepared by MG‐C and AR‐M, GR, and JAE critically revised the manuscript. Financial support was obtained by GR and AR‐M.

## Supporting information


**Fig. S1.** MicroR661 expression levels in colon cancer cell lines. Expression levels of miR661 are normalized to CCD841 primary colon cancer cell line. Primary cancer cell lines analyzed in the study: CCD18‐Co and CCD841. Colon cancer cell lines analyzed in the study: DLD1, SW620 and HT29.

**Fig. S2.** l‐lactate quantification.

**Fig. S3.** SW620‐miR661 stable cell line.

**Fig. S4.** Luciferase assays.

**Table S1.** Clinical and histopathological characteristics of stage II and stage III CC patients included in the study.

**Table S2.** Primers used for quantitative real‐time PCR.
