# Supplementary figures and images for: MicroRNA‐661 modulates redox and metabolic homeostasis in colon cancer
Source: Mol Oncol. 2017 Nov 6;11(12):1768–87. doi: 10.1002/1878-0261.12142 (PMC5709620; doi:10.1002/1878-0261.12142)

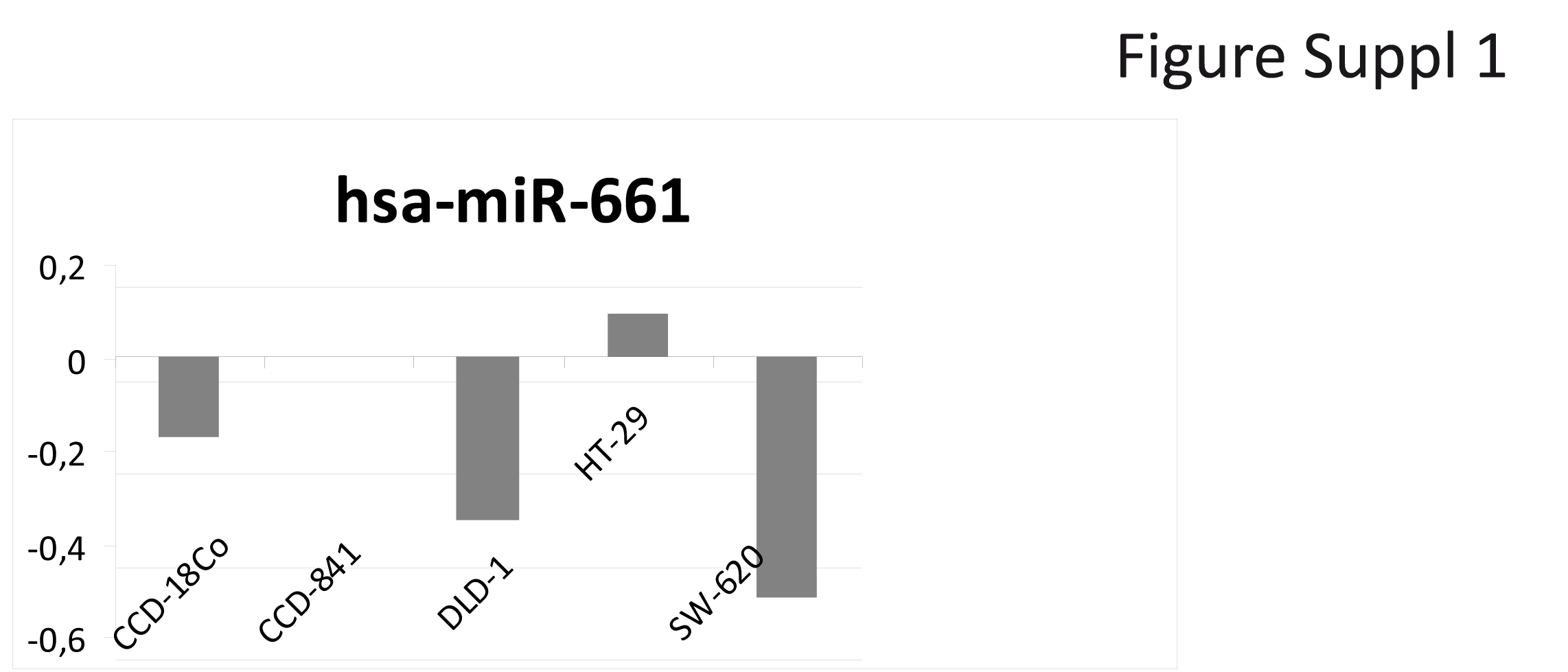

Supplement: Supplementary file 1 — Fig. S1. MicroR661 expression levels in colon cancer cell lines. Expression levels of miR661 are normalized to CCD841 primary colon cancer cell line. Primary cancer cell lines analyzed in the study: CCD18‐Co and CCD841. Colon cancer cell lines analyzed in the study: DLD1, SW620 and HT29. [file MOL2-11-1768-s001.tif]

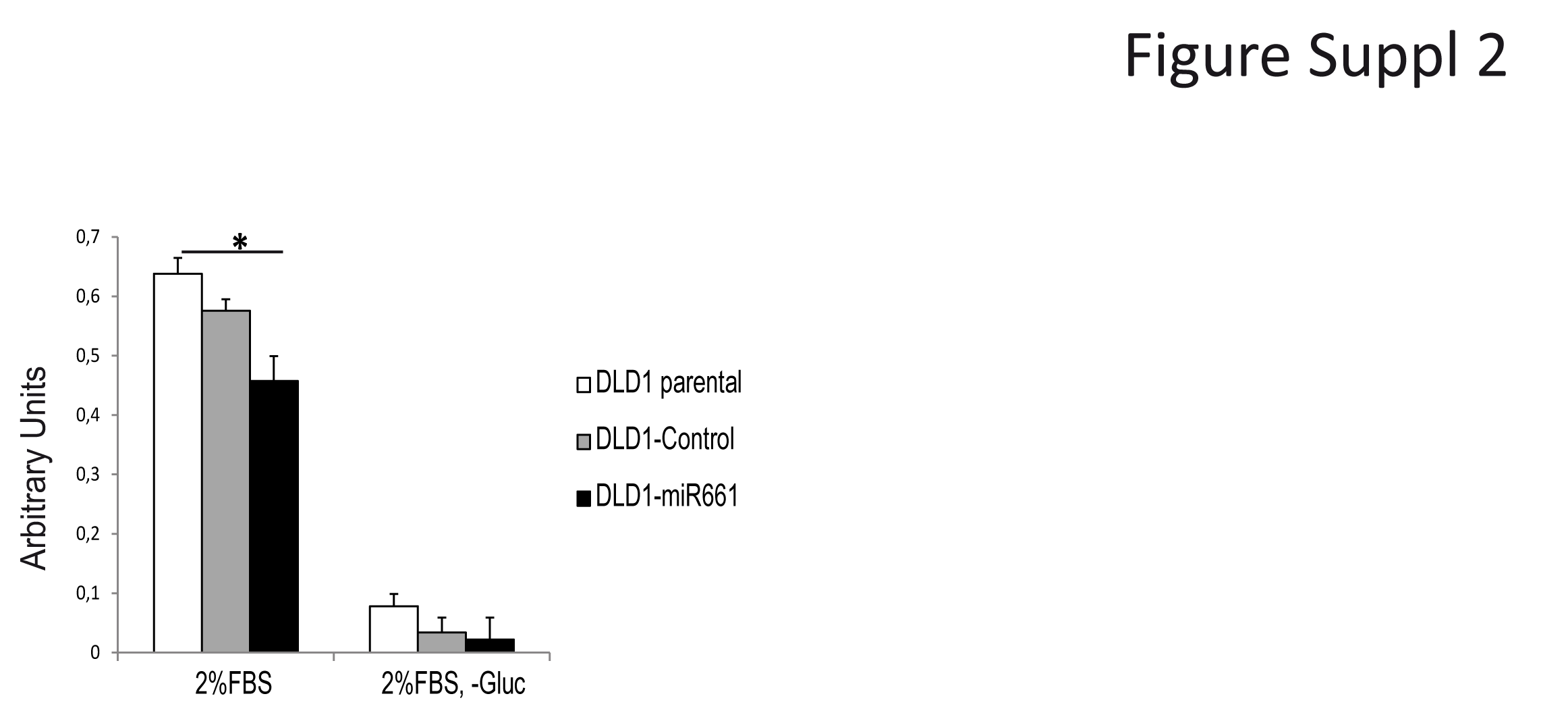

Supplement: Supplementary file 2 — Fig. S2. l‐lactate quantification. [file MOL2-11-1768-s002.tif]

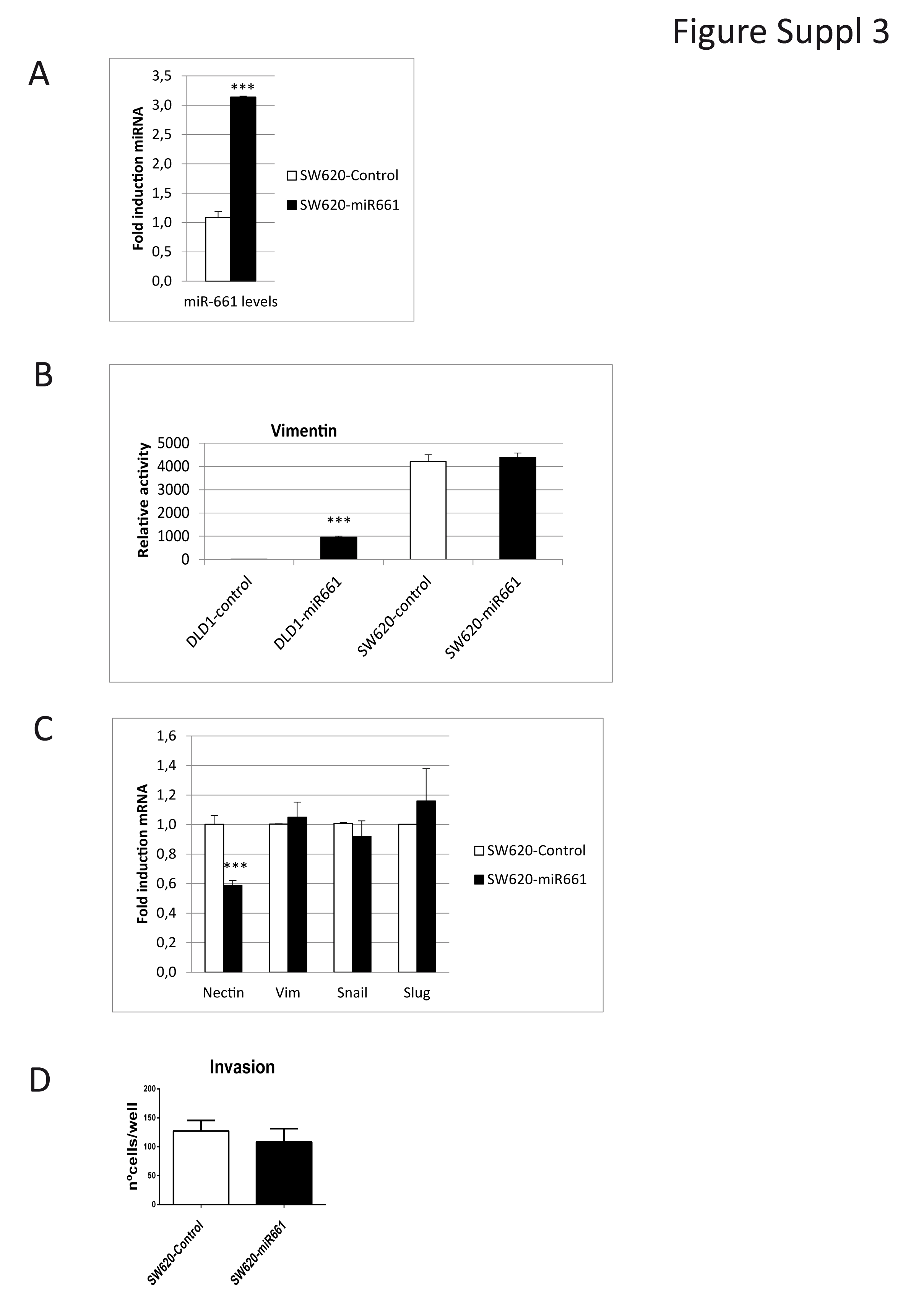

Supplement: Supplementary file 3 — Fig. S3. SW620‐miR661 stable cell line. [file MOL2-11-1768-s003.tif]

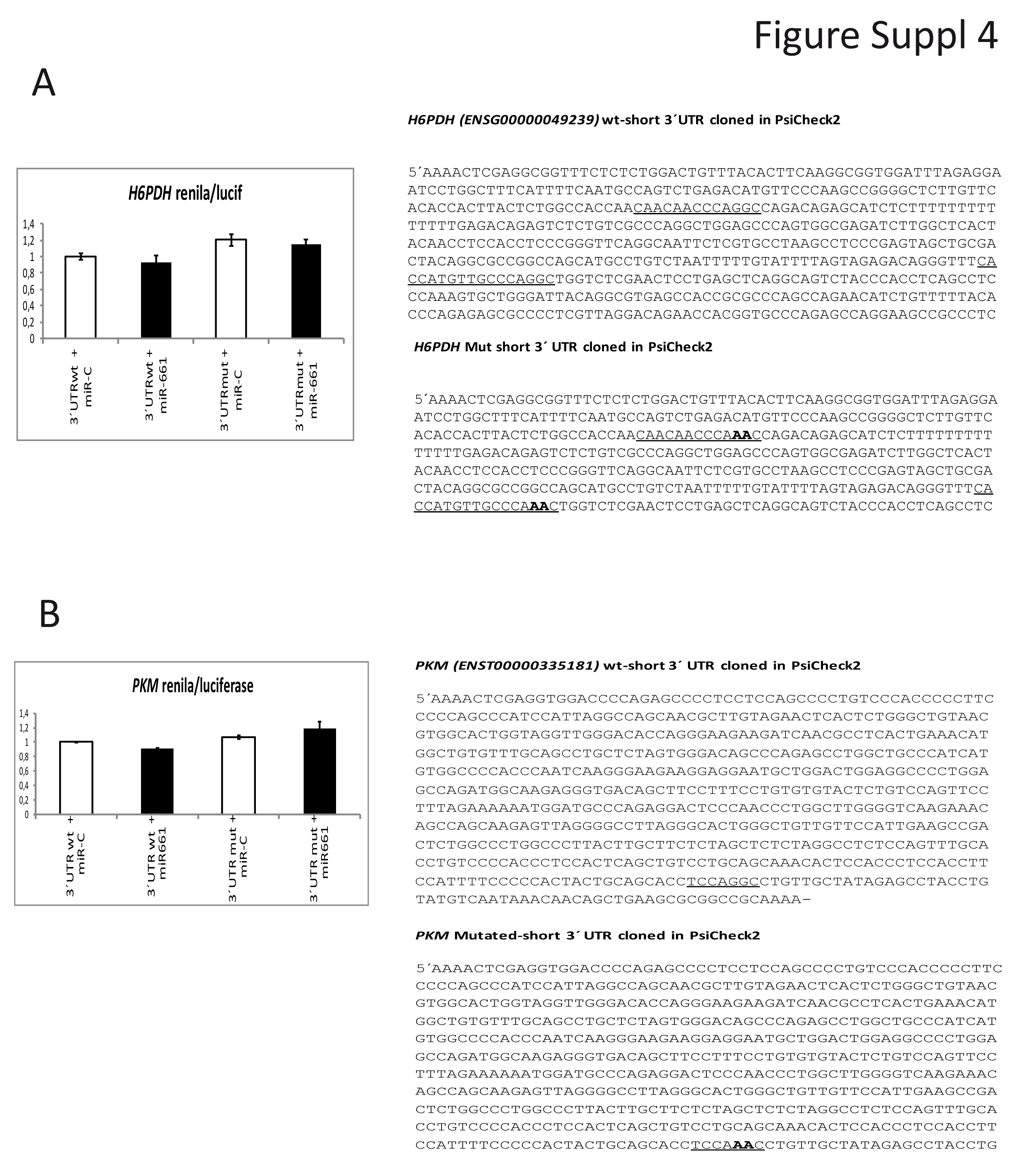

Supplement: Supplementary file 4 — Fig. S4. Luciferase assays. [file MOL2-11-1768-s004.tif]

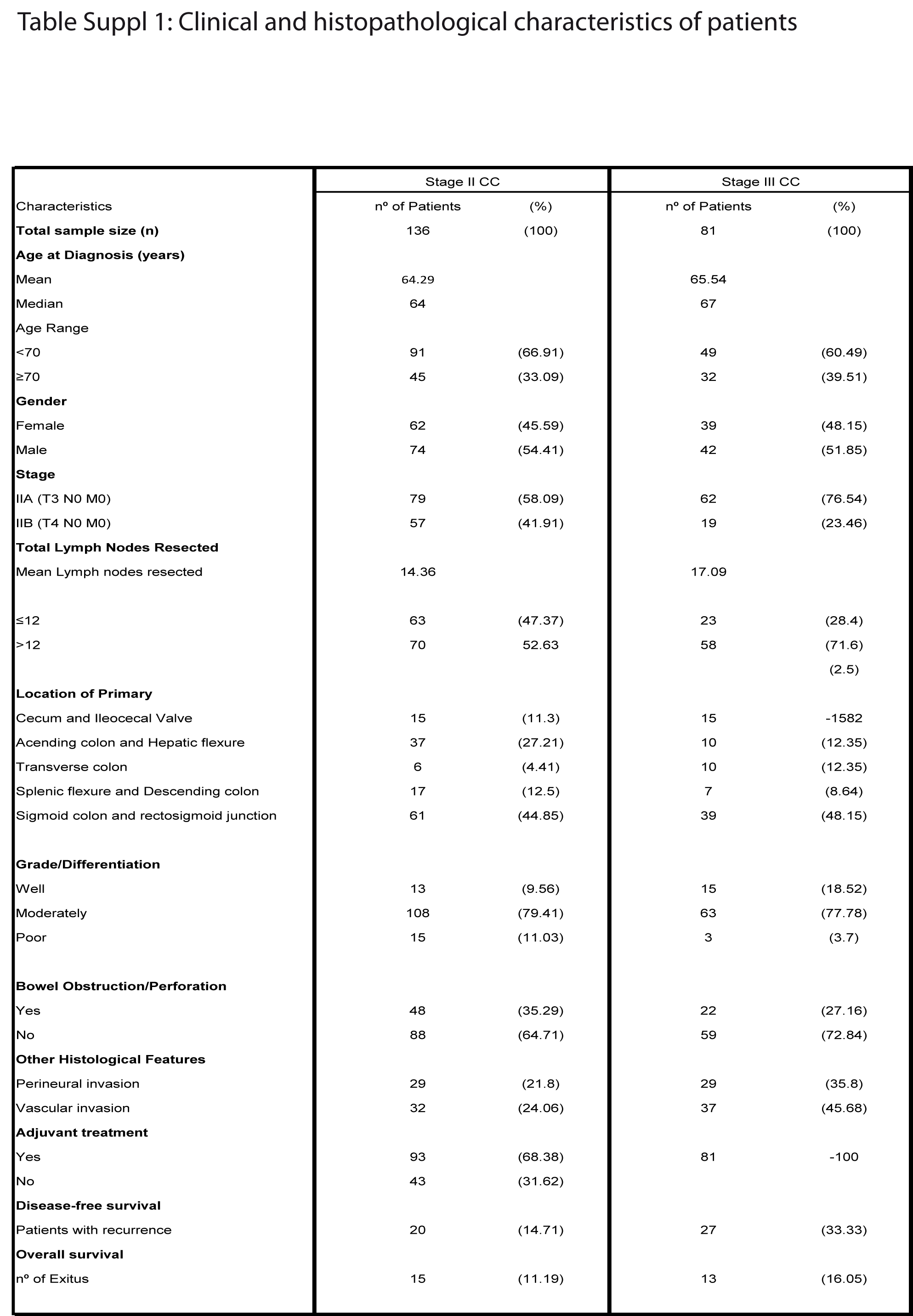

Supplement: Supplementary file 5 — Table S1. Clinical and histopathological characteristics of stage II and stage III CC patients included in the study. [file MOL2-11-1768-s005.tif]

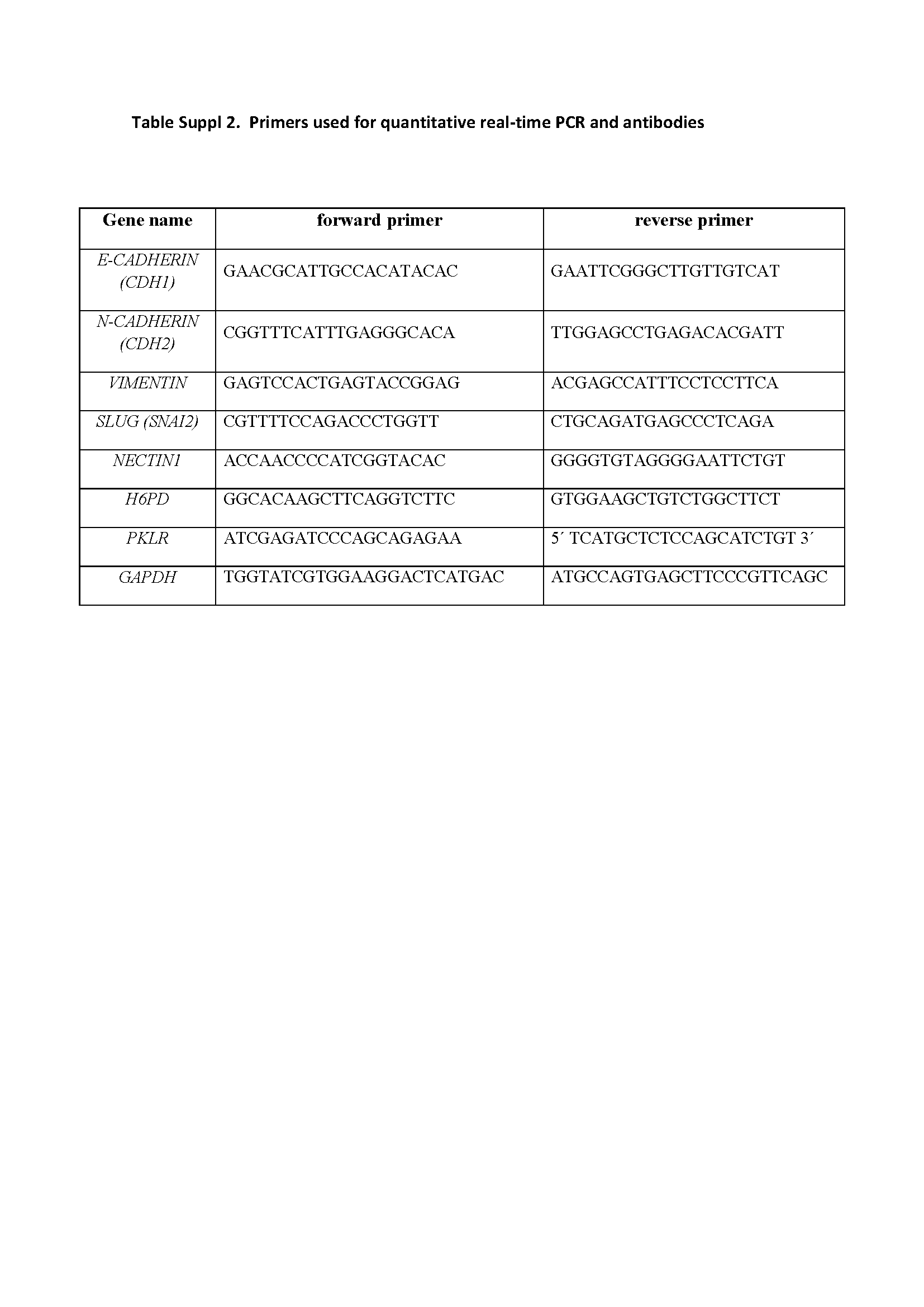

Supplement: Supplementary file 6 — Table S2. Primers used for quantitative real‐time PCR. [file MOL2-11-1768-s006.tiff]
